# Characterizing ABC-Transporter Substrate-Likeness Using a Clean-Slate Genetic Background

**DOI:** 10.3389/fphar.2019.00448

**Published:** 2019-04-25

**Authors:** Artem Sokolov, Stephanie Ashenden, Nil Sahin, Richard Lewis, Nurdan Erdem, Elif Ozaltan, Andreas Bender, Frederick P. Roth, Murat Cokol

**Affiliations:** ^1^Laboratory of Systems Pharmacology, Harvard Medical School, Boston, MA, United States; ^2^Centre for Molecular Informatics, Department of Chemistry, University of Cambridge, Cambridge, United Kingdom; ^3^Discovery Sciences, IMed Biotech Unit, AstraZeneca R&D, Cambridge, United Kingdom; ^4^Faculty of Engineering and Natural Sciences, Sabancı University, Istanbul, Turkey; ^5^Donnelly Centre, University of Toronto, Toronto, ON, Canada; ^6^Department of Molecular Genetics and Computer Science, University of Toronto, Toronto, ON, Canada; ^7^Lunenfeld-Tanenbaum Research Institute, Mt. Sinai Hospital, Canadian Institute for Advanced Research, Toronto, ON, Canada; ^8^Axcella Health, Cambridge, MA, United States

**Keywords:** ABC transport protein, cheminformatics, membrane transport, machine learning, drug structure

## Abstract

Mutations in ATP Binding Cassette (ABC)-transporter genes can have major effects on the bioavailability and toxicity of the drugs that are ABC-transporter substrates. Consequently, methods to predict if a drug is an ABC-transporter substrate are useful for drug development. Such methods traditionally relied on literature curated collections of ABC-transporter dependent membrane transfer assays. Here, we used a single large-scale dataset of 376 drugs with relative efficacy on an engineered yeast strain with all ABC-transporter genes deleted (ABC-16), to explore the relationship between a drug’s chemical structure and ABC-transporter substrate-likeness. We represented a drug’s chemical structure by an array of substructure keys and explored several machine learning methods to predict the drug’s efficacy in an ABC-16 yeast strain. Gradient-Boosted Random Forest models outperformed all other methods with an AUC of 0.723. We prospectively validated the model using new experimental data and found significant agreement with predictions. Our analysis expands the previously reported chemical substructures associated with ABC-transporter substrates and provides an alternative means to investigate ABC-transporter substrate-likeness.

## Introduction

ATP Binding Cassette (ABC)-transporters are membrane proteins used for the transfer of a variety of substrates across the cell membranes (Dean et al., 2001; Locher, 2016). These proteins are intensively studied due to their importance in several diseases (Borst and Elferink, 2002). For example, mutations in the CFTR chloride channel, which is encoded by ABCC7 gene, result in abnormal solute transportation in lungs and cause cystic fibrosis in humans (Guggino and Stanton, 2006). Another classic example is P-glycoprotein, encoded by ABCB1, for which mutations causing overexpression can result in multidrug resistance in cancer cells (Juliano and Ling, 1976).

ATP Binding Cassette-transporters are ubiquitous in all kingdoms of life (Szollosi et al., 2018). These membrane transporters have an ABC, which induces a structural change for the channel to open upon ATP hydrolysis (Jones and George, 2004). Small molecules or drugs may interact with ABC-transporters in two contexts. First, they may be ABC-transporter substrates, which are exported from the cell. If a drug is an ABC-transporter substrate, a mutation in the ABC-transporter genes may affect the bioavailability of that drug (Wu and V Ambudkar, 2014). Second, a drug may be an ABC-transporter inhibitor. These drugs can modulate the activity of ABC-transporters themselves, altering the intracellular concentration of compounds that are substrates of ABC-transporters and frequently cause drug-drug interactions (Zhou, 2008). For example, when P-glycoprotein inhibitors are combined with P-glycoprotein substrates, the increased bioavailability of the substrate may lead to serious side effects (Montanari and Ecker, 2015). Therefore, understanding both substrates and inhibitors of ABC-transporters is of extreme medical importance.

Experimental measurement of ABC-transporter/substrate relationships requires labor-intensive experiments involving the transport of a molecule across a monolayer of cells overexpressing ABC-transporters, rhodamine-123/calcein-AM fluorescence assays or flow cytometry (Adachi et al., 2001; Haynes et al., 2018). While such assays have been adopted as *de facto* standards in measuring ABC-transporter/substrate relationship, their cost precludes high-throughput screens. Previous computational studies on ABC-transporter/substrate relationship has relied on carefully curated collections of published data (Wang et al., 2011; Hazai et al., 2013). For example, a recently published dataset reported 822 ABC-transporter substrate/non-substrate molecules, curated from 517 published papers (Li et al., 2014). Authors used naive Bayesian classifiers on this dataset to explore the physicochemical and structural properties of ABC-transporter substrates. While these studies pave the way for a better understanding of ABC-transporters, the aggregated data may lead to inconsistencies due to different experimental setups in various labs (Montanari and Ecker, 2015). Transporter annotations are highly dependent on experimental factors, which may not be fully captured by database annotations. In addition, the use of aggregated data complicates the choice of prospective validation experiments for computational methodologies. Therefore, understanding of ABC-transporter substrates will benefit from a large-scale dataset where all the measurements are collected in a coherent fashion according to a common experimental protocol.

A recent study reported a strategy to delete a large set of genes in the yeast *Saccharomyces cerevisiae* and replace each with a Green Fluorescent Protein-expressing gene (GFP) (Suzuki et al., 2011). Using this strategy, the authors generated an “ABC-16 green monster” strain, in which all 16 *S. cerevisiae* ABC-transporters implicated in multi-drug resistance have been replaced with a GFP gene. This ABC-16 strain was tested against 376 drugs from the NIH Clinical Collection, which comprises compounds previously used in human clinical trials and covers a wide array of structure and target space. The authors reported that 31% of the drugs tested were more efficacious against the ABC-16 strain in comparison with the parental yeast strain (Suzuki et al., 2011). Such drugs are likely exported from the cell via ABC-transporters, and now achieve a higher intracellular concentration when the ABC-transporters are missing.

In our study, we revisited the dataset above to investigate whether drug efficacy in the clean-slate ABC-16 strain can be predicted from chemical structure properties of drugs. We define the compounds that have increased efficacy against the ABC-16 strain as “ABC-transport substrates.” We used the information for 376 compounds provided by the aforementioned screen as training. Our study identifies substructures associated with ABC-transporter substrates and derives a prediction model of substrate-likeness based on the presence/absence of these substructures. In addition, we conducted prospective validation experiments for 24 additional compounds and demonstrated success in predicting drug efficacy. Our study provides proof-of-concept that the yeast ABC-16 strain is a valuable model for exploring ABC-transport substrate specificity.

## Results

### Training Data Encapsulates the Presence of Chemical Substructures and Drug’s Efficacy Against the ABC-16 Strain

**FIGURE 1:**
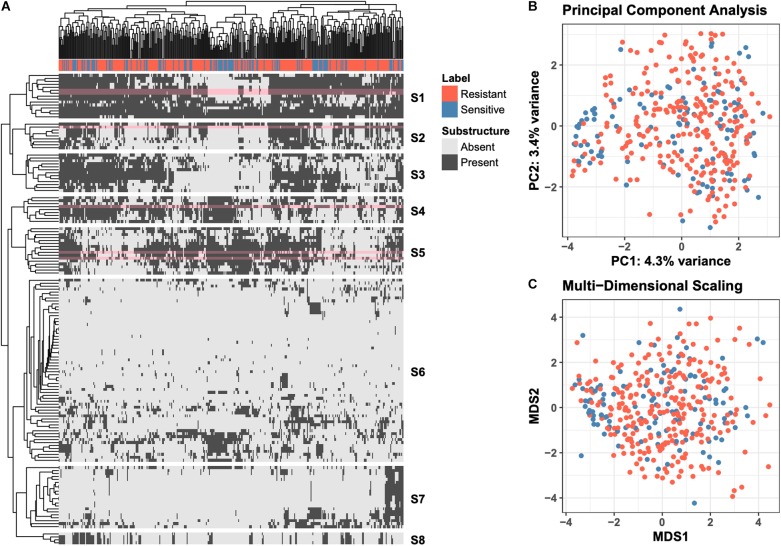
Overview of the raw data. **(A)** Clustered heatmap of 149 substructure features (rows) computed across 376 drugs (columns). Individual entries in the heatmap denote presence or absence of a particular MACCS fingerprint in the corresponding drug. Orange labels indicate drugs that are more efficacious against ABC-16 yeast strain than the parental strain. Optimal leaf reordering was applied to both rows and columns in an effort to reveal additional structure in the data that may not be observed from the default ordering. The six substructures (rows) revisited in more detail in Figure 2 are highlighted in pink. **(B)** Projection of the raw data onto the first two principal components. The amount of variance explained by each component is displayed in the axis label. **(C)** Projection of the raw data onto the first two components of Multi-Dimensional Scaling. The coloring of points is consistent across all three panels.

Molecular ACCess System (MACCS) keys define a set of 166 chemical substructures that are often found in small molecule drugs (Durant et al., 2002). For each of the 376 drugs used in the “green monster” study by Suzuki et al. (2011), we generated MACCS-key binary profiles; each entry in the profile indicates if the corresponding substructure is found in the drug’s chemical structure. The MACCS-key profiles of all 376 compounds are shown as a heatmap in Figure 1A, where rows correspond to MACCS keys and columns to drugs. The heatmap is hierarchically clustered with leaf-order optimization (Bar-Joseph et al., 2003) for improved visual clarity. An indicator of whether a drug is more efficacious against the ABC-16 strain is shown at the top of the heatmap. To provide additional exploratory view of the data, we also project raw high-dimensional data into two 2-D subspaces. We explore a linear projection via Principal Component Analysis (PCA), as well as a non-linear projection via Multi-Dimensional Scaling (MDS). These are presented in Figure 1B,C, respectively.

The data views presented in Figure 1 fall under the umbrella of *unsupervised learning*, where the computations do not explicitly incorporate the efficacy labels. Such views allow for general exploration of patterns in the data, which we observe do not align with the resistant/sensitive delineation. This motivates the need to employ *supervised learning* methods to explicitly model the relationship between a drug’s chemical structure and its relative efficacy against the ABC-16 strain.

### Enrichment of Substructures Provides an Indicator of Efficacy Against the ABC-16 Strain

**FIGURE 2:**
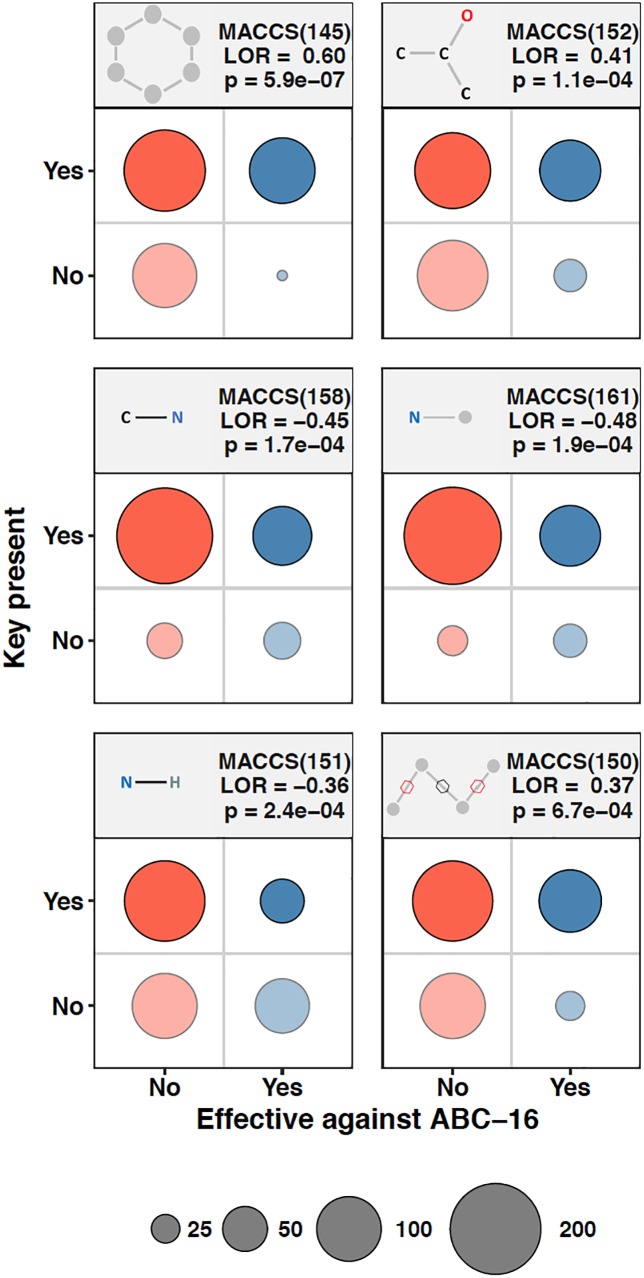
Chemical substructures significantly enriched or depleted in drugs that are effective against ABC-16. 2 × 2 contingency tables relating relative efficacy against ABC-16 and presence of substructure are given for six substructures. The number of drugs in each quadrant are represented by circle size. Also shown are the log-odds ratios (LOR) and the associated *p*-values calculated using Fisher’s Exact Test. When adjusted by Benjamini-Hochberg procedure, the six substructures shown exhibited FDR <5%.

We asked whether the presence of molecular fingerprints in a drug’s chemical structure can be used to make inference about the effectiveness of that drug against the ABC-16 strain. As a preliminary step to assess the amount of predictive power in individual MACCS keys, we composed contingency tables quantifying the presence of a given MACCS fingerprint and whether the corresponding drugs were more efficacious against the ABC-16 than the parental strain. Fisher’s Exact test was then applied to each contingency table to assess the relationship between the two variables. Because we applied Fisher’s Exact test to each of 166 keys, we adjust our significance estimates for multiple hypothesis testing. We applied the Benjamini-Hochberg procedure to estimate False Discovery Rate (FDR) from the set of *p*-values obtained from individual tests. Six MACCS keys had an FDR below 5%. These fingerprints, whose presence or absence was most closely associated with the corresponding drug’s relative efficacy in the ABC-16 strain, are highlighted in Figure 2. Figure 2 suggests that drugs with increased efficacy against ABC-16 strain are significantly enriched for six-atom ring structures [MACCS(145)]. This is consistent with an observation that five of the 15 substructures previously identified as indicators of P-glycoprotein substrate-likeness also included a six-atom ring structures (Li et al., 2014). In addition, we observed that a carbon atom which is bound to two carbons and one oxygen [MACCS(152)], or two non-rings connected by a ring bond [MACCS(150)] were also enriched in drugs with relative efficacy against the ABC-16 strain. Conversely, C-N, N-H, and N-^∗^ substructures are often absent among drugs with increased efficacy against ABC-16 strain.

While the above univariate approach of looking at one MACCS fingerprint at a time provides some evidence of predictive power, we expect that additional power can be gained by constructing machine learning models that incorporate information about the presence of multiple fingerprints simultaneously.

### Cross-Validation Performance Reveals Gradient-Boosted Machines as the Most Accurate Method for Predicting Relative Efficacy in ABC-16 Strain

We considered a panel of linear and non-linear machine learning methods. This panel included k-nearest neighbors (k-NN), regularized logistic regression, support vector machines (SVM), gradient-boosted random forest machines (GBM), and artificial neural networks (NNet). Each method was used to train a model that predicted whether a drug was more efficacious in the ABC-16 strain relative to the parental strain using the presence of MACCS fingerprints as input features.

**FIGURE 3:**
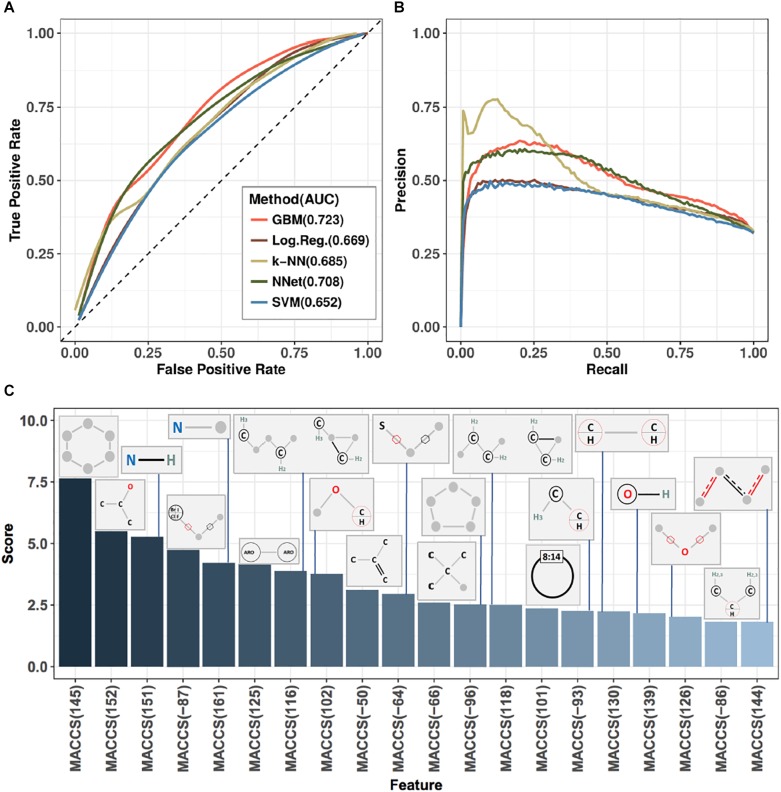
Performance metrics and analysis of substructure-based prediction of relative efficacy against ABC-16 yeast. **(A)** ROC curves estimating performance of five machine learning algorithms evaluated in ten-fold cross-validation. Each algorithm was trained over a grid of meta-parameter values, and the final predictions for any given test fold were computed by averaging predictions from individual models across this grid. Area under the curve (AUC) values are indicated next to method annotations. **(B)** Precision-recall curves for the five machine learning algorithms presented in panel **(A)**. GBM was selected as the top-performing method, based on AUC and accuracy metrics (Table 1). The method corresponds to the light red line in both panels **(A)** and **(B)**. **(C)** Feature importance scores from a Gradient Boosting Machine model trained on the entire training set using the best set of meta-parameter values (as determined from ten-fold cross-validation). Presented are the top 20 features and their associated MACCS fingerprints.

Ten-fold cross-validation was used to assess performance of predictors (see section “Materials and Methods”), with results shown in Figure 3 and Table 1. Figure 3 displays receiver-operator characteristic (ROC) and precision-recall curves (panels A and B, respectively) for all five methods, while Table 1 presents summary performance metrics. We observe that non-linear methods (k-NN, GBM, and NNet) outperformed linear methods (logistic regression and SVM), with gradient-boosted machines achieving a slight performance edge (overall AUC = 0.723, accuracy = 0.729) over NNet (overall AUC = 0.708, accuracy = 0.723) and k-nearest neighbors (overall AUC = 0.685, accuracy = 0.713). This is an expected trend as non-linear methods are able to capture more complex relationships between the presence and absence of individual MACCS keys than their linear counterparts. The accuracy values we obtained are slightly higher than those of previously reported models for predicting ABC-transporter substrates (∼0.72 compared to ∼0.70) (Aniceto et al., 2016) but lower than accuracy of models that focus specifically on P-glycoprotein substrates (∼0.72 compared to ∼0.83) (Li et al., 2014). This suggests that the difficulty of the prediction task may vary from one ABC-transporter to the next.

**Table 1:** Cross-validation performance metrics for five machine learning methods trained to predict ABC-16 relative efficacy.

Method	TP	TN	FP	FN	Precision	Recall	Accuracy	AUC
GBM	42	232	25	77	0.627	0.353	0.729	0.723
NNet	61	211	46	58	0.570	0.513	0.723	0.708
k-NN	40	228	29	79	0.580	0.336	0.713	0.685
Log.Reg.	49	203	54	70	0.476	0.412	0.670	0.669
SVM	62	195	62	57	0.500	0.521	0.684	0.652


*TP = True positive, TN = True negative, FP = False positive, FN = False negative.*

After assessing the performance of all methods through cross-validation, we trained a single final model on all drugs in the training set, using GBM and the best set of its meta-parameter values. GBM belongs to the class of Random Forest methods, which are defined as ensembles of simple predictors called “decision trees” (Natekin and Knoll, 2013). Each decision tree effectively asks “Is a particular molecular substructure present in a given drug?” and, depending on the answer to that question, either assigns a substrate/non-substrate label or routes the decision to the next question. Classical random forests construct decision trees by learning which features (i.e., substructures) best discriminate substrates from non-substrates on a random subset of data. GBM introduces a key concept called boosting, where each subsequent tree learns to predict the error of the currently constructed forest. In other words, the first decision tree learns to predict the labels directly, the second focuses on the drugs that were misclassified by the first tree, the third tree places additional focus on those drugs that were incorrectly classified by the first two trees, and so on.

Our choice of GBM was guided by its higher overall performance relative to the other methods, which is in line with recent studies successfully using GBM for ADMET predictions (Lei et al., 2017b, 3935–3953; Lei et al., 2017a, 2407–2421). An important property of GBM is that it tends to be conservative, as indicated by its higher precision, but lower recall values compared to other methods (Table 1). While it achieves the highest overall AUC and accuracy, GBM produces more false negatives than other methods, suggesting that the threshold for making substrate calls based on classifier output can be lowered. We revisit this property when discussing results on prospective validation data.

The final model was interrogated for feature importance scores by asking how well the GBM model performs when the values of a given feature are shuffled (see section “Materials and Methods”). This allowed us to identify the set of MACCS fingerprints with the highest predictive power. The top 20 fingerprints, their corresponding importance scores and substructure schematics are presented in Figure 3C. We observe that four of the six MACCS keys from Figure 2 were also among the most important features in the GBM model. The other two keys [MACCS(158) and MACCS(150)] were not among the 20 most important features in the model. This suggests that, while being informative in a univariate view, they carry redundant information when considered in concert with other features. Conversely, the MACCS(-87) feature—identified by GBM to be important—had a limited ability to stratify relative efficacy on the ABC-16 strain (log-odds ratio = 0.28, FDR = 0.13) by itself. Thus, the machine learning method was able to exploit features that were only useful in concert with other features.

The orthogonality of information provided by each feature can be judged by feature importance scores presented in Figure 3C. Features that get assigned high scores are deemed by the model to be jointly important for making accurate predictions. For example, MACCS(145) and MACCS(152) have univariate predictive power and both receive high importance scores, suggesting that they provide complementary information. On the other hand, MACCS(158), while being predictive in a univariate setting, does not appear among the top-scoring features in Figure 3C, suggesting that the information captured by MACCS(158) is redundant. Therefore, features are not entirely orthogonal and they add predictive value based on their importance.

### New Data Is Collected *in vitro* to Assess ABC-16 Efficacy of 24 Drugs Not Considered Previously

**FIGURE 4:**
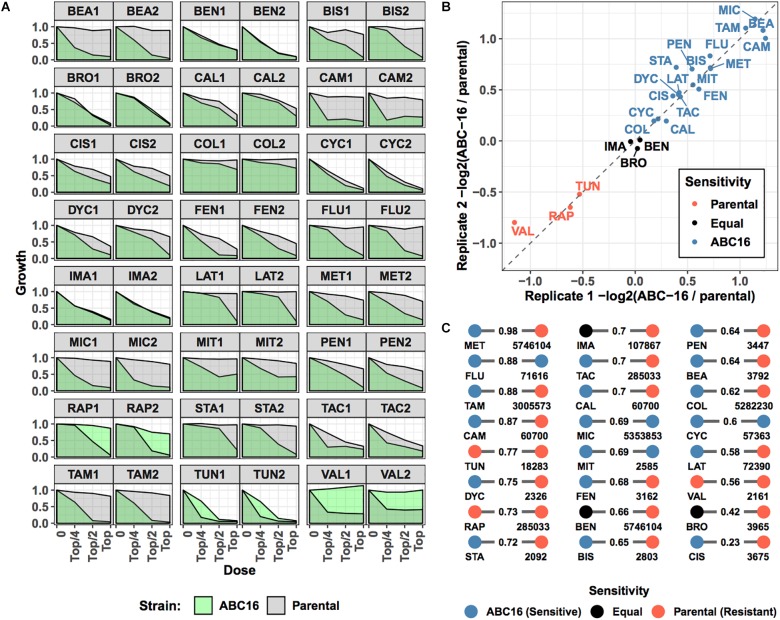
Overview of prospective validation dataset. Three-letter abbreviations for each drug are given in Table 2. **(A)** For 24 new drugs, dose-response experiments were carried out for parental and ABC-16 yeast strains, in duplicate. The drug doses in each experiment are two-fold dilutions starting from a top concentration shown in Table 2. Dose-responses for ABC-16 or parental yeast strains are shown in green and gray, respectively. **(B)** For each experiment, relative efficacy against the ABC-16 yeast is calculated by taking the –log2 of the ratio of the area under dose-response for ABC-16 yeast with the area under the dose-response of the parental strain. This score is zero if ABC-16 and parental yeast strains are equally sensitive. The score is negative or positive if ABC-16 yeast is more resistant or sensitive, respectively. As shown, the relative efficacy scores for the two replicates are highly concordant. The drugs with less, equal or more efficacy to ABC-16 compared to wild type yeast are shown in orange, black or blue, respectively. **(C)** Tanimoto similarity between prospective validation and training datasets, presented as a bipartite graph. For each drug in the prospective validation set (left nodes), we identified its closest neighbor in the training set (right nodes, annotated with PubChem IDs). Nodes are colored according to relative efficacy against ABC-16 yeast with Tanimoto similarity scores displayed as edge weights.

We next sought to validate our final model on new, previously unseen data. To do so, we considered 24 drugs that are not in the training set (Table 2). These 24 compounds were chosen from available compounds in our laboratory that (i) inhibit wild-type yeast (antifungal) and (ii) were not among 376 drugs in the training set. Eleven of these drugs were previously screened for their pairwise interactions (Benomyl, Bromopyruvate, Calyculin A, Dyclonine, Fenpropimorph, Latrunculin B, Pentamidine, Rapamycin, Staurosporine, Tacrolimus, and Tunicamycin) (Cokol et al., 2011, 2014). Six additional antimicrobials (Cycloheximide, Beauvericin, Colchicine, Fluconazole, Miconazole, and Valinomycin) and seven anti-cancer drugs (Bisantrene, Camptothecin, Cisplatin, Imatinib, Methotrexate, Mitoxantron, and Tamoxifen) were included to provide a large data set with sufficient power to test our GBM-based ABC-transporter substrate prediction method. We grew ABC-16 and parental strains treated in three doses of each drug, in biological duplicates. The top concentration of each drug was chosen to fully inhibit the growth of one of the two strains (see section “Materials and Methods”). We measured the growth in each condition and used the area under the dose-response curve as the growth metric for each strain in each experiment. Visual inspection showed that fluconazole, miconazole, beauvericin, camptothecin, mitomycin, and tamoxifen were inert against the parental strain at doses enough to inhibit the growth of the ABC-16 (Figure 4A). In contrast, ABC-16 strain was resistant to rapamycin, tunicamycin, and valinomycin.

**Table 2:** Name, abbreviation, PubChem ID, and top dose for each of the 24 drugs used for the prospective validation experiments.

Drug	Abbreviation	PubChem ID	Top dose (μg/ml)
Beauvericin	BEA	3007984	25
Benomyl	BEN	28780	28
Bisantrene	BIS	5351322	105
Bromopyruvate	BRO	70684	15
Calyculin A	CAL	5311365	2.1
Camptothecin	CAM	24360	175
Cisplatin	CIS	441203	700
Colchicine	COL	6167	8000
Cycloheximide	CYC	6197	0.37
Dyclonine	DYC	3180	10
Fenpropimorph	FEN	93365	10
Fluconazole	FLU	3365	38
Imatinib	IMA	5291	1950
Latrunculin B	LAT	3892	1.4
Methotrexate	MET	4112	220
Miconazole	MIC	4189	0.07
Mitoxantrone	MIT	4212	270
Pentamidine	PEN	4735	3.85
Rapamycin	RAP	5284616	0.0013
Staurosporine	STA	44259	4.4
Tacrolimus	TAC	445643	3.6
Tamoxifen	TAM	2733526	23
Tunicamycin	TUN	6433557	0.083
Valinomycin	VAL	441139	1000


To make a more quantitative comparison, we defined the relative efficacy of each drug as -log2( ABC-16 growth/parental strain growth). This efficacy measure is 0 if the two strains are equally sensitive to the drug. A positive or negative efficacy value indicates increased or decreased efficacy against the ABC-16 strain, respectively (see section “Materials and Methods” for details). Relative efficacy for drugs significantly correlated among two replicates (Figure 4B). We used the average of two measurements as each drug’s ABC-dependent efficacy.

Finally, we considered the difficulty of predicting the newly collected efficacy measures by computing Tanimoto similarity between each of the 24 drugs in our validation set and training data. Figure 4C shows the closest match in the training set for each validation drug, highlighting the difficulty of the prediction task: a large number of closest matches have opposite labels. For example, Methotrexate (MET) has 0.98 Tanimoto similarity to glutamic acid (PubChem ID: 5746104) but is more efficacious against the ABC-16 strain, while glutamic acid is more efficacious against the parental strain. Such label discrepancy among similar compounds presents a major challenge for machine learning methods.

We make the raw data collected for prospective validation, a wrangled copy of the training data, and all code are publicly available at https://labsyspharm.github.io/ABCmonster/.

### Prospective Validation Confirms That the GBM Model Is Able to Infer Relative Efficacy of New Drugs Against the ABC-16 Strain

**FIGURE 5:**
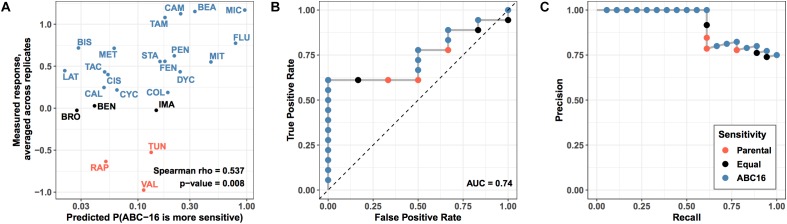
Performance of prospective validation of substructure-based prediction of relative efficacy against ABC-16 yeast. **(A)** For each drug in test set, the X axis shows the predicted probability of ABC-16 sensitivity, and the Y axis shows the relative efficacy scores –log2(ABC-16 growth/parental strain growth). These scores are higher for compounds to which ABC-16 is sensitive. Substructure-based relative efficacy predictions significantly correlated with the mean relative efficacy scores from two replicates (Spearman rho = 0.54, permutation test *p*-value = 0.007), accurately capturing ABC-16 yeast’s sensitivity to fluconazole and miconazole, and ABC-16 yeast’s resistance to tunicamycin, rapamycin and valinomycin. **(B)** ROC curve showing predictor performance on the validation data, where drugs with higher efficacy against ABC-16 yeast are treated as “positive” examples, while drugs with lower or equal efficacy are treated as “negative” examples. **(C)** The matching precision-recall curve. All points across all panels are colored consistently with Figure 4, with drugs that have less, equal or more efficacy to ABC-16 compared to wild type yeast being shown in orange, black and blue, respectively.

We asked how well the final GBM model is able to predict efficacy measured in our validation set of drugs. Using the model, we generated probability for each drug’s increased relative efficacy against the ABC-16 yeast strain and matched the predictions against measured values (Figure 5). Despite the prediction task difficulty imposed by mismatched labels among highly similar drugs, we observed a significant Spearman correlation between predicted and observed efficacy (Spearman rho = 0.54, permutation test *p*-value = 0.007) (Figure 5A). The correlation allows us to compare predictor output to true measurements without placing a threshold at a fixed probability value. In this way, we are effectively asking whether the predictor correctly ranks substrates above non-substrates. This is in direct parallel to AUC, which is widely regarded as a more robust performance metric than accuracy, because it does not rely on a fixed threshold to make predictions (Ling et al., 2003).

Next, we binarized the experimental measurements, treating drugs that were more efficacious in the ABC-16 strain as “positive” examples. Likewise, drugs with equal or lower efficacy in the ABC-16 strain than in the parental strain were assigned the “negative” label. The binary labels then allowed us to construct ROC and Precision-Recall curves, which are displayed in Figure 5, panels B and C, respectively. We observed an AUC value of 0.74 and very high precision at recall values of over 50%. The high precision in particular demonstrates the value of the GBM model when only a limited number of substrate candidates is requested.

Lastly, we considered the question of placing a threshold on the probabilistic output of GBM to assign substrate/inhibitor calls to individual predictions. While 0.5 may seem like a natural choice for such a threshold, it would result in a large number of false negatives, as Miconazole and Fluconazole would be the only two drugs to get correctly identified as substrates. This is consistent with our earlier observation that GBM is overly conservative, as highlighted in our discussion of cross-validation results (Table 1). For this reason and based on our examination of the validation data results, we suggest lowering the threshold to 0.15 when assigning substrate/inhibitor labels to new drugs. Due to high precision of the GBM method, lowering the threshold should be robust with respect to false positives. Under the threshold of 0.15, GBM will correctly identify 11 out of 18 substrates in the given validation set, with no false positives.

We believe the reason GBM is able to overcome the task difficulty has to do with which features it deems important for prediction (Figure 3C). For example, Camptothecin and Topotecan HCL (PubChem ID: 60700) have Tanimoto similarity of 0.87 (Figure 4C) but different labels. One of the molecular fingerprints the two drugs differ in is MACCS(-93), GBM correctly scores Camptothecin above Topotecan HCL, because it considers MACCS(-93) to be among its top 20 most-predictive features (Figure 3C). Likewise, Beauvericin gets scored higher than Itopride HCL (PubChem ID: 3792) because it contains N-H substructure, MACCS(151), which is deemed by the GBM model to be the third most predictive feature.

## Discussion

It has been more than 40 years since mutations in P-glycoprotein were linked to multidrug resistance in cancer. It is now known that P-glycoprotein is a member of the ABC-transporter family of proteins, which selectively transport molecules out of the cell using energy. However, it is also understood that the same molecule can be transported by more than one ABC-transporter, and each ABC-transporter can transport more than one type of molecule. These two factors complicate the study of ABC-transporter substrate specificity, necessitating computational models to study this phenomenon. All such models constructed to date used collections of small scale data for the learning task. Majority of the studies toward this aim focused on P-glycoprotein – substrate relationship. Our study uses a large-scale experimental data set that was published in a single article, and provides a means to investigate the ABC-transporter substrate-likeness for a compound when all ABC-transporters are considered. Using a cheminformatics based framework, we detected chemical substructures that are over or under-represented in ABC-transporter substrates. We used substructure profiles of drugs to train a machine learning method to predict ABC-transporter substrates. N-^∗^ substructure was a particularly good univariate predictor in our model. A previous study has shown that one of the best predictors for ABC-transporter relationship was N-branched substructures (Li et al., 2014). To the best of our understanding, a mechanistic explanation for the presence of N-^∗^ and ABC-transporter substrate-likeness is lacking. Strong univariate predictors like N-^∗^ can be starting points for explaining how an ABC-transporter selectively transports multiple compounds with different molecular structures. The fact that a linear array of substructure presence can be used for ABC-transporter substrate-likeness prediction is promising. Future studies may consider the long-distance relationships between substructures and shed light on the scaffolds of chemical structures with high ABC-transporter substrate-likeness.

Interestingly, all -mycins in the test set (Rapamycin, Tunicamycin, and Valinomycin) were less efficacious against the ABC-16 strain. This is fully consistent with the four -mycins present in the training set (dactinomycin, clarithromycin, kitasamycin, and oligomycin c), which are also less efficacious against the ABC-16 strain. Therefore, the poor efficacy of –mycins against ABC-16 is a reproducible and frequent phenomenon, which is learned by our framework. These compounds may provide another departing point for a mechanistic exploration of the many-to-many relationship between ABC-transporters and their substrates.

Chemogenomic interactions are defined as a surprising change in a drug’s effect in a given genetic background (Giaever et al., 2004; Jones and Bunnage, 2017). Numerous previous studies have shown that chemogenomic interactions between drugs and single gene deletions are rare (Hillenmeyer et al., 2008; Nichols et al., 2011). This is often explained by robustness in cellular machinery, where overlapping functions of genes allow compensations by genes with similar functions. A corollary of this explanation is that if all genes pertaining to a certain cellular function are deleted, chemogenomic interactions will be more frequent. Our observations are in agreement with this, as chemogenomic interactions were frequent when all genes that encode “proteins to transport some chemicals out of the cell using ATP” are deleted. With the recent advances in genomic editing methods such as CRISPR, groups of genes can be readily deleted (Cong et al., 2013; Adli, 2018) - similar to the ABC-16 strain used in this study, which was painstakingly generated using rounds of mate selection (Suzuki et al., 2012). The use of cells in which genes with overlapping functions are deleted may prove useful for illuminating the cellular mechanisms targeted by drugs.

We proposed the ABC-16 yeast strain as a clean-slate genetic model for studying ABC-transport substrate specificity. Using homogeneous data collected with a consistent experimental protocol, we trained a machine learning model to predict drug relative efficacy in this strain, allowing us to identify which compounds are ABC-transport substrates based directly on their chemical substructures. The model was then experimentally validated on a new panel of drugs, demonstrating its generalizability to previously unseen data. Being able to correctly identify ABC-transport substrates is a precursor step toward understanding the bioavailability of these molecules in polypharmacological studies and combination therapies. The presented study acts as a proof-of-concept that such a step can be performed *in silico*, potentially alleviating some of the costs associated with expensive compound screens. The information about MACCS key fingerprints that we highlighted as being most predictive can be utilized during early development in future drug design.

## Materials and Methods

### Generating and Assessing Predictive Models

The SMILES key representation for each compound was extracted from PubChem (Kim et al., 2016). SMILES keys for each compound was processed using the open-source cheminformatics tool RDKit v2015 (Landrum, 2013) to check the presence of a predefined set of 166 MACCS structural keys. In this way, the structure of each compound was converted to a numeric vector, which we referred as structural compound fingerprints. These vectors and their associated labels (ABC-16 sensitive or not) were used as the input for the machine learning experiments detailed below.

Ten-fold cross-validation was used to assess method performance. Each cross-validation run was repeated 100 times to assess robustness with respect to random number generation (used for splitting data into folds, bootstrap sampling in random forests, initializing neural network weights, etc.). In each run, we iterated through the set of all drugs, withholding a tenth at a time. At each iteration, a model was trained on the non-withheld data and subsequently used to score the withheld subset. The training set has a class imbalance, where about 1/3 of the samples were ABC-16 Sensitive and 2/3 were ABC-16 Resistant. We ensured that the class imbalance was properly captured in each fold while splitting the data for cross-validation. Since each fold was an accurate representation of the data as a whole, no additional weighting was imposed. Performance estimated through cross-validation was further averaged across a grid of meta-parameter values. These included the number of neighbors in k-NN, the number and depth of trees in GBM, the number of hidden units in NNet, and the L1 (LASSO) and L2 (ridge) penalties for regularized logistic regression and SVM.

Marginalizing out meta-parameter values by averaging across performance values is distinct from the more traditional approach of selecting a single, usually the best-performing, set of values in each fold. By averaging across a grid of values instead, we incorporate a measure of robustness when comparing performance across the five methods. All training and model evaluation was performed in R using the *caret* package (Kuhn, 2008).

To determine variable importance in a GBM model, we computed out-of-bag prediction accuracy for each tree (Friedman, 2001). A second accuracy value is computed after permuting the values of that variable across the entire training set. The difference between the two performance measures is then averaged across all trees in the random forest and normalized by the standard error. Finally, the importance scores from individual boosting iterations are summed together.

### Strain Growth Measurements

Rich media (YPD) consisted of 1 g yeast extract, 2 g peptone, 5 mL 40% glucose per 100 mL of media. Drug solutions are prepared in YPD for 4 different dosages. The minimum of (i) concentration that inhibits the growth of the parental yeast strain and (ii) concentration that inhibits the growth of ABC-16 strain was chosen as the Minimum Inhibitory Concentration (MIC) of each drug. 50 × MIC concentration of each drug was prepared in DMSO. In four vials, 90 μL of YPD was mixed with (i) 10 μL 50 × MIC, (ii) 5 μL 50 × MIC + 5 μL DMSO, (iii) 2.5 μL 50 × MIC + 7.5 μL DMSO, and (iv) 10 μL DMSO. 20 μL of these drug solutions were transferred to 96-well plates. Overnight cultures of parental and green monster strains were grown in 2 mL YPD with 100 μL cells from glycerol stocks, at 30°C temperature and 200RPM shaking. These cultures were diluted to OD600 = 0.01 in YPD and 80 μL of the diluted culture was added on each well. After the addition of cells, each well has 2% DMSO and their respective drug dose. Plates were sealed and shaken at 30°C for 15 h with OD600 measurements at 15 min intervals, using Tecan Infinite F200 plate-readers. For each drug, we therefore collected growth curves in three concentrations and one no drug control condition. We used the area under the growth curve as the growth metric in each concentration and normalized each growth to no drug condition to construct the dose-response curves shown in Figure 4A. The area under each dose-response was used as the concentration independent growth metric for the compound, with low and high values indicating sensitivity and resistance, respectively. Growth of ABC-16 strain was compared with the growth of wild-type strain to assess relative efficacy. (Growth ABC-16/growth wild-type) was -log2 transformed for visual clarity; compounds with 0, negative or positive have equal, lower or higher relative efficacy against ABC-16 strain, respectively.

## Author Contributions

SA, NE, and EO prepared the data. AS, RL, and MC analyzed the data. NS conducted the experiments. AS, AB, FR and MC wrote the manuscript.

## Conflict of Interest Statement

MC is employed by Axcella Health. The remaining authors declare that the research was conducted in the absence of any commercial or financial relationships that could be construed as a potential conflict of interest.
